# Evidence of Seasonal Variation in Body Color in Adults of the Parasitoid *Cirrospilus pictus* (Hymenoptera: Eulophidae) in Sicily, Italy

**DOI:** 10.3390/insects14010090

**Published:** 2023-01-13

**Authors:** Mirella Lo Pinto, Salvatore Guarino, Alfonso Agrò

**Affiliations:** 1 Department of Agricultural, Food and Forest Sciences (SAAF), University of Palermo, Viale delle Scienze, Building 5, 90128 Palermo, Italy; 2 Institute of Biosciences and Bioresources (IBBR), National Research Council of Italy (CNR), Corso Calatafimi 414, 90129 Palermo, Italy

**Keywords:** body color, parasitoid, *Phyllocnistis citrella*, morphology, indigenous natural enemies

## Abstract

**Simple Summary:**

Many studies highlighted that the body color variation in insects is an adaptation factor that preserves them from possible population reductions due to adverse conditions such as low temperatures. In this study, the coloration seasonal changes of *Cirrospilus pictus*, parasitoid of the citrus leafminer *Phyllocnistis citrella* were observed. Adults were obtained from field sampling carried out during four consecutive years and grouped in different classes depending on sex and color. Results highlighted a phenotypic pigmentation variation in head, thorax, gaster and legs of the adults. Individuals were yellow–green in summer months while having dark pigmentation in autumn and winter months. In both sexes a correlation between color patterns and seasonal temperatures was evident. These observations can contribute to the description of the intraspecific variability of this species, improving its identification.

**Abstract:**

As part of the studies on the morphological color variation of insects, a case study on the seasonal body color variation of *Cirrospilus pictus* (Nees) (Hymenoptera: Eulophidae: Eulophinae) parasitoid of leafminers is reported. Observations were made from January 2000 to December 2003 in north-western Sicily (Italy), in relation to sex, body regions of adults and seasonal periods. Wasps parasitizing *Phyllocnistis citrella* Stainton (Lepidoptera: Gracillariidae) were collected from organic citrus orchards (*Citrus limon* L., var. “Femminello zagara bianca” and “Femminello comune”). Adults were grouped in classes: yellow males, black males, yellow females, yellow–black females and black females. The results highlighted a phenotypic pigmentation variation in the head, thorax, gaster and legs of individuals influenced by the season of sampling. Adults were yellow–green in summer months, whereas individuals with dark pigmentation were found in autumn and winter months. A correlation between color patterns and seasonal temperatures was found for both females and males. This work provides a contribution to the description of the intraspecific variability of this species, improving its identification.

## 1. Introduction

Insects show a great variety of colors [1] that can have relevant biological functions such as thermoregulation [2], warning (aposematic) coloration [3] or mimicry [4], secondary sexual characters [5], and predator avoidance (crypsis and masquerade) [6]. Coloration can be due to structural colors (forms of surface and epidermal structures) or pigments (outer body layers) that selectively absorb, reflect, or scatter the light. Orange, red, yellow, and brown–black colors of the body observed in insects derive from pigments, while blue or green colors are often due to structural features [1]. Either the colors or the way they are arranged into patterns often vary among individuals of a species. For example, among Hymenoptera, many species of Scelioninae, Tetrastichinae and Eulophinae show a recurring color pattern of a black head, orange mesosoma, and black metasoma [7]. In several species of Scelioninae, variation between individuals of the same sex appears to be quite common, with the mesosoma varying from orange to entirely black [8,9]. Additionally, some Ichneumonidae species show intraspecific variation, for example, in females where the mesoscutum varies from entirely orange to almost entirely black [10]. In some apoids, either females or males often show intraspecific color variation [11]. In sawflies some species have males all black and females varying in color [12], some species (e.g., *Perreya tropica* Norton) have some males showing the mesoscutum orange while others the entire thorax and abdomen, and females with both the thorax and abdomen orange, but the dark wings [7]. In addition, among Chalcidoidea, *Cirrospilus vittatus* Walker shows an extensive color variation, with individuals ranging from almost completely dark metallic green or blue to completely yellow with no metallic markings [13].

The morphological diversity in the Chalcidoidea, as a function of the host, seasonal dimorphism and dichroism [14], complicates their classification. In fact, the taxonomy of the Chalcidoidea is overall based on the comparison of morphological features, but these can be related to fitness and strongly influenced by the environment and temperature [15]. Therefore, the differentiation of species based on variable morphological features whose real variance is unknown can lead to mistakes [16]. In addition, the morphological and biological diversity within many species complicates chalcidoid taxonomy. For example, different hosts cause the variation of progeny from a female to vary in seasonal dimorphism and dichroism [14].

Among chalcidoid parasitoids, *Cirrospilus pictus* (Nees) (Hymenoptera: Eulophidae: Eulophinae) is a species that shows a phenotypic variability in relation to pigments of the body. Normally its body is yellow–green, but sometimes it is darker in some parts. It shows considerable variation in size and body coloration in relation to dark spots and stripes. Indeed, it has been re-described by some authors and synonymies were published by Zhu and coworkers [17]. Here, we present a case study to provide a contribution to the description of the intraspecific variability of this species and improve its identification.

*Cirrospilus pictus* is one of the indigenous larval parasitoids found on *Phyllocnistis citrella* Stainton (Lepidoptera: Gracillariidae) in Sicily (Italy) since the summer of 1995 [18,19,20], the year in which the phytophagous appeared in this region [21]. This pest is active during the summer and autumn months, producing about 10 generations/year with a life-cycle of 15 days in the summer and 134 days in winter [22]. From June 1996, this eulophid has shown the highest incidence on the complex of living parasitoid species on *P. citrella*, being, in some periods of the year, the only species present on the leafminer [19,23]. *Cirrospilus pictus*, also, can develop as primary or secondary parasitoids of other lepidopterous, hymenopterous and coleopterous leafminers [24]. Since seasonal dichroism is known in many eulophids [14], we hypothesized that *C. pictus* can exhibit a variation of body colors depending on climatic factors, especially temperature.

In this study, we focused on adults of *C. pictus* obtained from *P. citrella* collected in different period of the year in order to detect the chromatic variation of the body, in relation to sex and body regions. The main objective of our research was to provide a tabulation of the color pattern in *C. pictus* developing on the citrus leafminer during the year to improve its identification. In addition, we investigated the correlation between color patterns and seasonal temperatures.

## 2. Materials and Methods

This study was carried out during four consecutive years, from January 2000 to December 2003, in six distinct locations belonging to organic citrus orchards, situated in areas of north-western Sicily (Italy) (latitude varies from 37°40′ to 38°04′ N, longitude varies from 12°35′ to 13°56′ E). 

Wasps of *C. pictus* were obtained from plants of *Citrus limon* L., var. “Femminello zagara bianca” and “Femminello comune”, infested by *Phyllocnistis citrella* Stainton (Lepidoptera: Gracillariidae). No insecticides were applied to the organic citrus orchards. In each location, 200 young citrus shoots (length 20 cm) were randomly collected every 7 days, placed in plastic bags, and brought to the laboratory of the SAAF Department of the University of Palermo (Italy). In the laboratory (23 ± 1 °C; 60 ± 5%; R.H., L:D 16:8 h), leaves were observed under a stereomicroscope, in order to detect parasitized hosts. Pre-imaginal individuals with their hosts were put singly into glass tubes (l = 7.5 cm Ø = 1 cm) sealed with wet cotton and containing a small amount of honey as food. All the test tubes, appropriately marked, were kept in a climatic chamber under the same environmental conditions previously mentioned and observed daily until emergence of parasitoid adults. Once emerged, parasitoid adults were counted, isolated and sexed. Adults were examined under the microscope, noting the pigmented parts of the body, such as head, thorax, gaster and legs, and grouped into chromatic classes. Color patterns were recorded in dorsal view and the diversity of yellow–black patterns of all the observed wasps was documented. The individuals were assigned to 5 chromatic classes: I = yellow females, II = yellow–black females, III = black females, IV = yellow males, V = black males, grouped according to the different colors of the parts of their body. An aliquot of adults of each class was sent for their identification to Dr. John LaSalle of the International Institute of Entomology, CAB International of London.

A regression analysis between the number of yellow wasps and mean temperature (climatic data were furnished by the Sicilian Informative Agrometereological System-SIAS) in the four years of observations was performed. A one-way ANOVA was used to evaluate the mean number of wasps belonging to the five chromatic classes detected in the different months. Finally, the chromatic variation between females and males was evaluated by a Student’s t-test. Statistical analyses were performed using Statistica 7.1 for Windows Package (Stat Soft Inc., Tulsa, OK, USA).

## 3. Results

**Figure 1:**
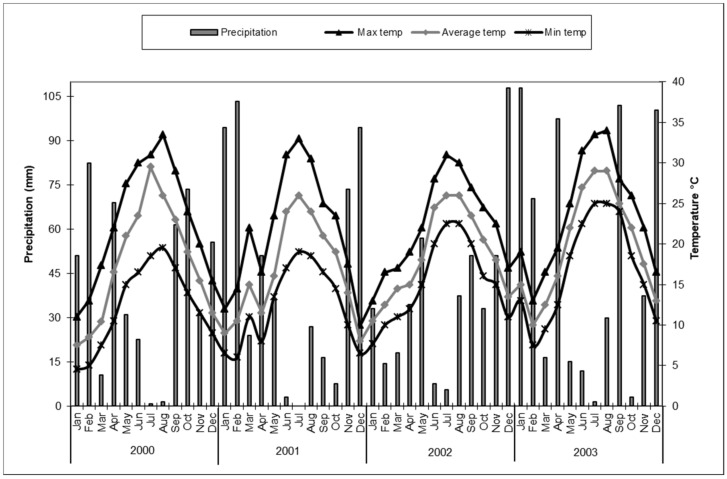
Total rainfall (mm) and mean temperature (°C) trends per month (SIAS) detected in the surveyed field of *Citrus* spp. in Sicily (Italy) from January 2000 to December 2003.

*Cirrospilus pictus* adults, either males or females, showed a chromatic variability depending on the month of the year in which they developed. In addition, the sexes differed in the color patterns. During the four years of study, the adults were obtained from June to February (period of main activity of the host *P. citrella*) and the color patterns observed always appeared similar in each year. The rainfall and temperature reported during the period of the study are reported in Figure 1.

**Figure 2:**
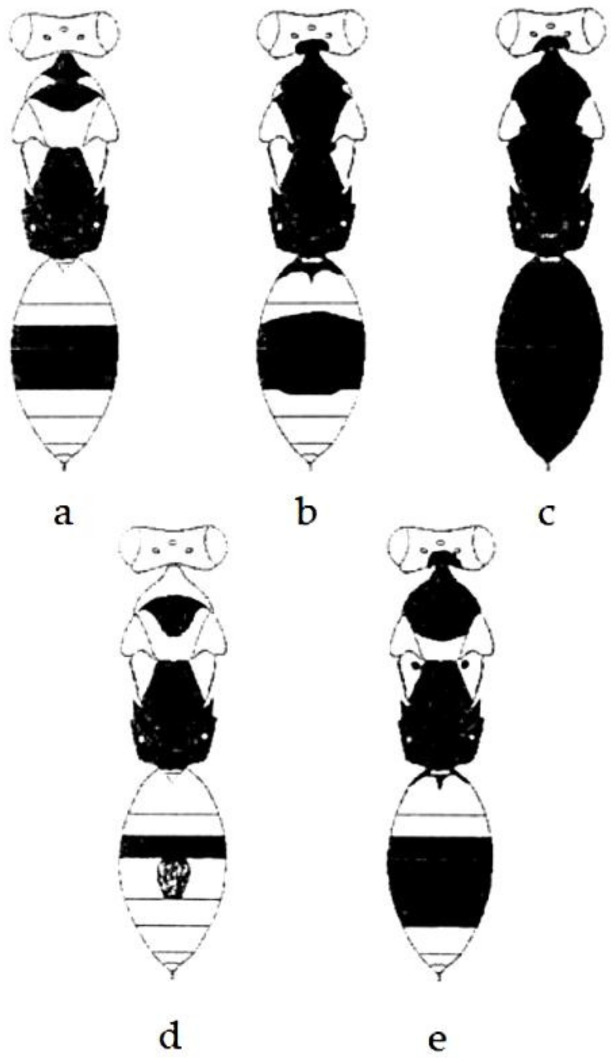
Scheme of chromatic variation of *C. pictus* body observed in the five classes: (**a**) class I = yellow females, (**b**) class II = yellow–black females, (**c**) class III = black females, (**d**) class IV = yellow males, (**e**) class V = black males.

**Figure 3:**
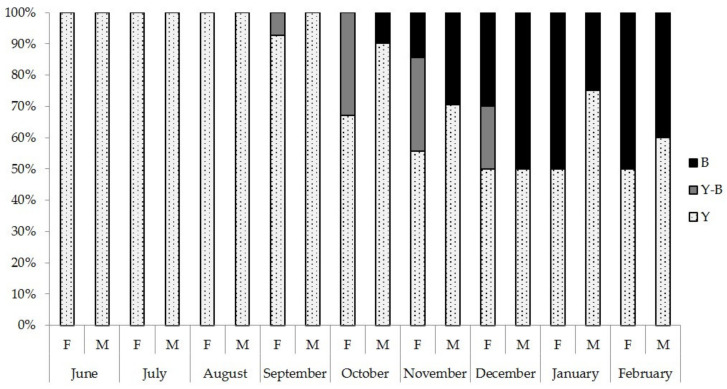
Monthly percentages of *Cirrospilus pictus* females (F) and males (M) belonging to classes I and IV = yellow (Y), II = yellow–black (Y–B), and III and V = black (B), averaged over the 4 years of observation (2000–2003) detected in the period of active infestation of *Phyllocnistis citrella*.

Assuming that, generally, the body of adults is yellow–green, a partial or total darkening of some parts of the body was observed. In total, five color patterns were observed in this species, three patterns in females and two in males. Specifically, the parts subject to change in color were the occiput, pronotum, mesoscutum, axilles, tergites and tibiae of middle legs, with differences between males and females (Table 1 and Figure 2). The percentage of adults belonging to chromatic classes, detected in the months of years in which infestations of *P. citrella* were active, is reported in Table 2 and Figure 3.

**Figure 4:**
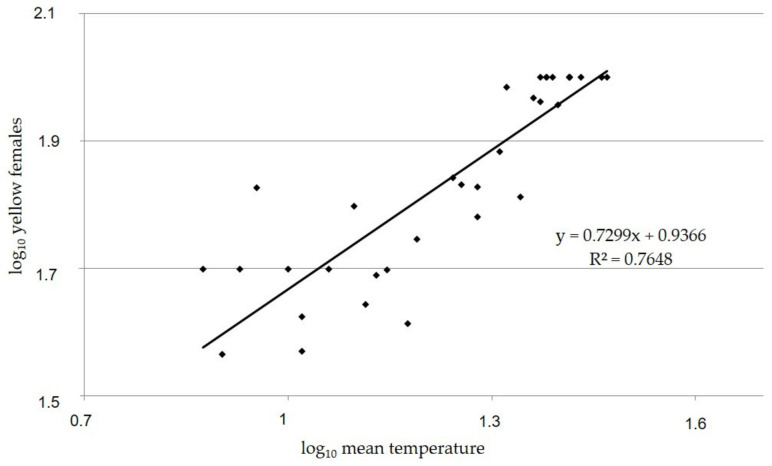
Linear regression between number of yellow females of *Cirrospilus pictus* and log of mean temperature (°C) (SIAS) detected in the surveyed field of *Citrus* spp. in Sicily (Italy) from January 2000 to December 2003.

**Figure 5:**
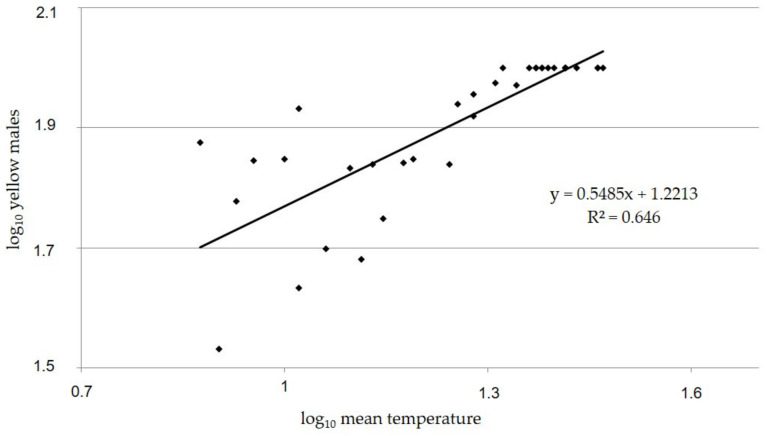
Linear regression between number of yellow males of *Cirrospilus pictus* and log of mean temperature (°C) (SIAS) detected in the surveyed field of *Citrus* spp. in Sicily (Italy) from January 2000 to December 2003.

Our observations confirmed that *C. pictus* varies greatly in coloration. Results highlighted that yellow individuals (classes I and IV) were the only phenotype present in the summer months and their percentage decreased to a minimum of 50% in the autumn and winter months. Black males (class V) began to appear from October up to February, whereas females showed yellow–black individuals (class II) from September to December and black individuals (class III) from November to February. In November and December, the three classes (I, II and III) of females coexisted. The infestation of host *P. citrella* was not detected in the areas of observation from March to May; therefore, no parasitoids were recorded in this period. The percentage of yellow wasps ranged between 50% (winter months) and 100% (summer months) for both females and males. In the period of appearance, the percentage of yellow–black females ranged from a minimum of 7.2% (September) to a maximum of 32.8% (October). The percentage of black wasps ranged between 14.3% (November) and 50% (December) for females, and between 9.75% (October) and 50% (December) for males. Generally, the dark color of black wasps on the thorax has some metallic shine. Males show a dark patch on the pronotum and a large transverse brown stripe on the gaster, and often a black spot on the yellow middle tibiae. This spot may also be present in females, but not dark. Statistical analysis showed significant differences among the number of wasps belonging to the five classes detected in the different months for both females (F = 34.87, df = 8, *p* < 0.001) and males (F = 22.41, df = 8, *p* < 0.001). Significant differences in the chromatic variation were also found between females and males (t = 1.68, df = 35, *p* < 0.001). This phenotypic variability dependent on season was confirmed by the regression analysis that showed a significant correlation between the number of yellow wasps and temperature (SIAS data) for both females (F = 110.5, df = 0.07, R^2^ = 0.76, *p* < 0.001) (Figure 4) and males (F = 62.4, df = 0.07, R^2^ = 0.64, *p* < 0.001) (Figure 5).

## 4. Discussion

Variation in body coloration is well documented in insects and generally has a large genetic component [25,26,27]. The results presented in this paper report a case of seasonal chromatic variation of the body of *C. pictus* observed in both female and male individuals. Seasonal dichroism is known in Eulophidae [14,28], and several other families of Hymenoptera also show chromatic variation [29,30,31,32]. For example, during the spring, individuals of *Eulophus larvarum* L. [33] and *Atoposomoidea unipunctata* (Nees) [34] show coloration different from that of other seasons; some species belonging to the genus *Cirrospilus*, *Sympiesis*, *Eulophus* and *Olynx* exhibit seasonal dichromism [35]. The seasonal color variation of *C. pictus* found in this study is also reported in other areas of the Mediterranean basin, such as in Spain [36].

The chromatic variation can be affected by several factors, such as temperature, humidity [29,37], host species [14,28,29,31,38,39,40,41,42], size or physiological condition of the host [43,44]. This color variation is considered an adaptive function based on the thermal budget hypothesis, i.e., a darker color absorbs more solar radiation, visible or infrared, at low temperatures [15,45,46,47,48,49]. In fact, the dark individuals generally reach higher body temperatures and warm up more quickly than paler individuals [50,51,52]. As the darker individuals absorb solar radiation more effectively than the paler ones [53], and reach higher body temperatures, they can exhibit higher activity levels in colder climates, leading to better pest control in such conditions [50,54]. Moreover, darker forms may have the thermal optimum at a lower level than light-colored ones [55]. Many authors state that variation in thermal capacity is likely to have important implications for individual fitness, affecting activity period, energy budget, escape capability, dispersal, mating success, and fecundity [50,52,56,57,58]. This aspect appears to be affected by natural selection related to climatic factors that leads to genetically based variation in the body color of individuals showing different activity levels in different areas [59,60,61,62].

Our results showed that, in the case of *C. pictus*, only some parts of body were subjected to chromatic variation and these differed between sexes. In agreement, Zhu and colleagues [17] reported that *C. pictus* females from different regions show some varieties in the coloration of the ocellar triangle, occiput, pronotum, axilla, hind coxae, and gaster. The coloration of some parts of the body due to temperature is also reported in other parasitoid species, for example in *Pnigalio soemius* (Walker) in which this parameter determines a strong influence on the pigmentation of the gastral tergites and other body parts, with both the tergites and tarsi tending appearing darker at lower temperatures [39]. Similarly, in the case of the braconid *Bracon hebetor* (Say), the body color of adults observed appears black at 15–18 °C, yellowish-black at 25 °C, and a yellow color at temperatures over 30 °C [63]. Another example is given by the aphid parasitoid *Trioxys utilis* Van de Bosh (Hymenoptera: Braconidae) that presents white-colored cocoons during warm-weather conditions and dark brown cocoons during cold-weather conditions [64]. It is likely that such morphological adaptations have helped parasitoids to survive extreme temperatures and could enable them to survive in a climate change conditions [65].

In our results, the dark pigmentation was sharper in females than in males, likely in dependence on the temperature. A lesser effect of temperature on the chromatic variation of males has been also reported for other insects [15,16,66,67]. In agreement with our results, Sundby [37] reported that females of *C. pictus* obtained from *Phyllocnistis labyrintella* Bierk showed a variation based on a darker pigmentation of thorax, not detected in males, but in our observations the darkening can affect almost the entire body of females.

In terms of seasonal distribution, the most widespread patterns were yellow wasps (classes I and IV), followed by black (classes III and V) and then yellow–black (class II) wasps.

The detected chromatic classes of wasps have been similar for each year of the studied period and strongly dependent on climatic conditions. In particular, the increase in the temperature caused an increase in individuals belonging to classes I and IV (yellow wasps).

## 5. Conclusions

The observations conducted in this study confirm the hypothesis of a color variation of the body of *C. pictus* depending on the seasonal temperature. In particular, light individuals are more frequent in spring–summer and dark ones in autumn–winter, showing a correlation between color patterns and seasonal temperature. These results suggest an ecological adaptation to climatic conditions and could help in the identification of the species. In addition, the application of this knowledge could be useful to improve the suitability of natural enemies and their effectiveness in pest control.

## Figures and Tables

**Table 1:** Coloration of body parts detected in females of *Cirrospilus pictus* grouped in the assigned chromatic classes I = yellow females, II = yellow–black females, III = black females, IV = yellow males, V = black males. All classes are observed in dorsal view.

Body Regions	Anatomic Parts	Class I	Class II	Class III	Class IV	Class V
Head	occiput	yellow	black	black	yellow	black
Thorax	pronotum	partially black	almost totally black	black	yellow	black
	mesoscutum	partially black	more than half black	almost totally black	partially black	half black
Thorax Appendages	wings: axilles	yellow	black patch	black	yellow	black patch
	middle legs: tibiae	yellow	yellow	yellow	partially black in the middle	totally black in the middle
Gaster	tergite I	yellow	proximal black stripe	black	yellow	proximal small black stripe
	tergite II	yellow	partially black	black	yellow	yellow
	tergite III	black	black	black	black	black
	tergite IV	black	black	black	dark patch	black
	tergite V	yellow	partially black	black	yellow	black
	tergite VI	yellow	yellow	black	yellow	yellow
	tergite VII	yellow	yellow	black	yellow	yellow
	tergite VIII	yellow	yellow	black	yellow	yellow

**Table 2:** Percentage (mean ± SE) of *Cirrospilus pictus* wasps belonging to classes I and IV (yellow females and males) detected in the months of active infestation of *Phyllocnistis citrella* in 4 years (2000–2003).

Months	Females	Males
June	100 ± 0.0	100 ± 0.0
July	100 ± 0.0	100 ± 0.0
August	100 ± 0.0	100 ± 0.0
September	92.8 ± 2.6	100 ± 0.0
October	67.2 ± 6.7	90.2 ± 5.1
November	55.6 ± 8.7	70.5 ± 12.7
December	50.0 ± 14.0	50.0 ± 14.4
January	50.1 ± 12.0	75.0 ± 7.4
February	50.0 ± 10.5	60 ± 12.5

## Data Availability

The data are available on request to the authors.
